# Stigma of mental illness and cultural factors in Pacific Rim region: a systematic review

**DOI:** 10.1186/s12888-020-02991-5

**Published:** 2021-01-07

**Authors:** Mao-Sheng Ran, Brian J. Hall, Tin Tin Su, Benny Prawira, Matilde Breth-Petersen, Xu-Hong Li, Tian-Ming Zhang

**Affiliations:** 1 Department of Social Work and Social Administration, The University of Hong Kong, Hong Kong SAR, China; 2 Department of Psychology, The University of Macau, Macau SAR, China; 3 South East Asia Community Observatory (SEACO), Jeffrey Cheah School of Medicine and Health Sciences, Monash University, Selangor DE, Malaysia; 4 Into The Light Indonesia Suicide Prevention Community for Advocacy, Research and Education, Jakarta, Indonesia; 5 Sydney School of Public Health, University of Sydney, Sydney, Australia

**Keywords:** Mental illness, Stigma, Culture factors, PacificRim region, Intervention, Measurements

## Abstract

**Background:**

Although cultural factors play a crucial role in experience of stigma, there is scant review on the impact and importance of culture on stigma of mental illness across Pacific Rim Region. This study aims to investigate: 1) the cultural factors related to stigmatizing beliefs about mental illness in Pacific Rim region, and 2) culture-specific measures and interventions on stigma of mental illness.

**Methods:**

A systematic search of papers was conducted in the MEDLINE, Embase, CINAHL, Web of Science, PsycINFO, Scopus, Cochrane Library and Google scholar through January 2003 to April 2019.

**Results:**

Forty-one studies in Pacific Rim region which met the inclusion criteria were included in the study. The rate of stigma of mental illness (e.g., public stigma: from 25.4 to 85.2%) was relatively high in Pacific Rim region. Culture factors (e.g., Collectivism, Confucianism, face concern and familism, religion and supernatural beliefs) contributed to people’s stigmatizing behaviors and attitudes toward persons with mental illness, their relatives and mental health professionals. Certain measurements were developed and employed to assess different type of cultural factors related to stigma of mental illness.

**Conclusions:**

Cultural factors play an important role in influencing the rate and performance of stigma of mental illness. Further research on stigma of mental illness and culture-specific interventions to reduce the stigma should be conducted in the Pacific Rim region.

## Background

Stigma of mental illness, involving in stereotypes, prejudice and discrimination, has been viewed as a significant obstacle to the improving quality of life for people with mental disorder [1]. Numerous empirical findings show that stigma of mental illness is common among persons with mental illness throughout the world and results in a range of negative adversities [2–4]. Exposure of stigma, persons with mental illness may avoid treatment seeking, which contributes to the global treatment gap, and worsens outcomes in physical and mental domains [5]. Mental illness stigma also contributes to suicidal ideation in persons with mental illness, mediated by social isolation [6], secrecy and hopelessness, as well as anticipated discrimination [7].

Several types of stigma have been identified and illustrated by a substantial body of literature. In earlier studies, many researchers mainly focused on public stigma, also known as social and enacted stigma, which refers to general public negative reaction toward persons with mental illness [4, 8]. In the recent years, the focus has been shifted from public stigma to the subjective experience of stigmatized persons with mental illness, such as self-stigma, also called internalized stigma, to gain a better understanding of mental illness stigma process, given the fact that people with mental illness may internalize such stigmatizing beliefs from society, which they turns against themselves [2, 8] and may hinder their recovery [9].

A systematic review conducted by Ng (1997) [10] highlighted that persons with mental illness experience stigma in a multitude of societies and cultures. However, the manifestation of stigma may vary from a cultural perspective. Stigma of mental illness should be investigated and analyzed within its sociocultural context for the purpose of understanding its origins, meanings and effects. Surprisingly, although cultural factors play a crucial role in the experience of stigma which shapes attitudes, beliefs and values toward mental illness, there is scant literature focusing on the impact and importance of culture on stigma of mental illness [11].

Numerously previous studies ignore cultural and ethnic differences with regard to stigma [12]. There are few investigations of stigma of mental illness within the Pacific Rim region, an area of significant variance, including culture, religion, values, and mental health care systems [13]. There are developed and developing countries with eastern and western cultures in the Pacific Rim region which may differentially influence the stigma of mental illness. For example, the reason of stigma occurring varies between East Asians endorsing collectivist culture and Caucasian Americans endorsing individualist culture. Although intercultural relations and acculturation phenomena prevail in this region [14], there are few studies comparing the difference regarding the cultural factors on stigma in this region.

Understanding the relationship between cultural factors and stigma of mental illness in Pacific Rim region will be crucial for development of culture-specific anti-stigma interventions. Thus, the present review should be crucial for exploring the stigma of mental illness and cross-cultural characteristics in the Pacific Rim region. The present systematic review aimed to investigate: 1) the salient cultural values related to stigmatizing beliefs about mental illness in countries of the Pacific Rim, 2) specific measures for stigma of mental illness with a cultural perspective, and 3) anti-stigma interventions emphasizing cultural values in countries of the Pacific Rim region.

## Methods

### Search strategy and protocol registration

The following electronic databases were searched through January 2003 to April 2019: MEDLINE, Embase, CINAHL, Web of Science, PsycINFO, Scopus, Cochrane Library and Google scholar. Additionally, we checked reference lists of relevant articles and ran a search of relevant studies individually. The following keywords were used on screening titles and abstracts: (“mental illness” OR “mental disorders” OR “psychiatric disorders” OR “common mental disorders” OR “mental health”) AND (“stigma” OR “discrimination” OR “prejudice” OR “stereotype”) AND (“cultural values” OR “cultural beliefs” OR “face” OR “cultural characteristics”). The review was pre-registered at the PROSPERO (CRD42020138108). We have followed PRISMA reporting guidelines.

### Inclusion criteria and study selection

The reviewers (TMZ & XHL) screened and evaluated each title and abstract to identify relevant articles. Citations and abstracts were imported into Endnote X9. If a study was identified as relevant to the review, the reviewers accessed the full text article. English articles that were published and peer-reviewed were considered for this review. Studies were not restricted to a certain group of participants, stigma type, cultural factor, outcome and study design. However, if comparisons were conducted, it is necessary to demonstrate sufficient statistics. An article was selected for inclusion if it met all of the following criteria: 1) to be related to stigma of mental illness; 2) in the Pacific Rim; and 3) addressed culture factor. We employed a cautiously analysis to access the risk of bias using the Critical Appraisal Skills Program (CASP). The reviewers recorded “yes”, “no” or “can’t tell” in each question of appraisal checklists [15, 16]. In addition, a pilot assessment on 5 articles was undertaken separately by two reviewers to check the consistency, then we started the formal analysis. Studies were judged independently by two reviewers (TMZ & XHL) and those with an unacceptable risk of bias were excluded. Disagreements and uncertainties were discussed until reviewers reached consensus with a senior reviewer (MSR).

### Data extraction and study characteristics

After identifying studies for inclusion, reviewers extracted relevant data and developed a set of data extraction categories: (1) overview of stigma type, cultural values; (2) study characteristics including author, year, location of study (country), study type, stigma type, target population, measurements, salient cultural values and main findings. Additionally, the rates of stigma (e.g., self stigma and public stigma) reported in the results within quantitative studies were extracted directly for further analysis. After summarizing the results in the data extraction table, the reviewers extracted the data through a data collection tool that was developed by MSR and summarized the results into five broad categories by consensus of all reviewers, as follows: 1) cultural factors: cultural factors affecting stigma of mental illness, such as causal attributions, religion, value orientation, cultural norms, and beliefs; 2) stigma categories: stigma framework of Corrigan and Watson [1], by adding the other two types; 3) anti-stigma interventions: being categorized according to socio-ecological levels; 4) measurements: scales employed; and 5) rates of stigma: being extracted or calculated to generate the final number.

## Results

### Study selection

**Fig. 1:**
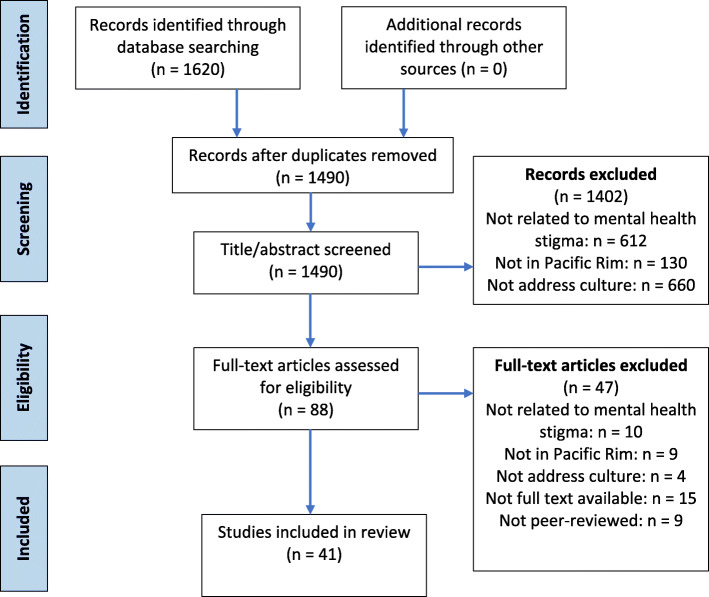
Procedure of the Review (identification, screening, eligibility and included)

Figure 1 shows the procedure of the review. The initial database search obtained 1490 published English-language articles after removing duplicates. The next stage of study selection involved a manuscript review of 88 articles. Some articles were excluded as they were not peer-reviewed (*n* = 9), not related to mental health stigma (*n* = 10), not in Pacific Rim (n = 9), not related to culture (*n* = 4), not full text available (*n* = 15). Articles that substantiating pilot assessment were also included. A total of 41 studies were included in the final review.

### Characteristics of the included studies

Table 1 shows the characteristics of reviewed studies. A total of 41 studies were identified, including 14 qualitative only studies, 19 quantitative only studies, 3 mixed-method studies and 5 theory studies. Among these, 13 studies were undertaken in the USA, 18 in Asia and 3 in Latin America. In addition, 5 studies were carried out across more than one continent. There were 3 studies focusing on intellectual disabilities specifically, 1 study on bipolar disorder, 1 study on substance use, 3 studies on depression, and 6 studies on schizophrenia. The majority of these studies focused on mental health and mental illness in general. 5 studies specifically targeted youths and students. 1 study had a teacher focus and 3 studies concerned mental health providers, the remaining concentrated on adults or non-specified job groups. Regarding types of stigma, 20 studies included public stigma, 13 studies involved self-stigma, 10 studies involved affiliate stigma and only 2 studies focused on professional stigma. Nine studies reported rates of stigma. Across all the studies included in the final review, specific intervention was poorly conducted, but many studies attempt to present the intervention and policy suggestion for alleviating stigma. Viewing the cultural factors, apart from 1 study interpreting multiple cultural factors, supernature beliefs and religion were reported on 9 studies, followed by face concern and familism reported on 29 studies, collectivism and Confucianism were reported by 8 studies, 3 studies addressed individualism.

**Table 1:** Characteristics of Reviewed Studies

Author, year	Study type	Target population (n)	Country/District	Cultural values	Stigma type	Measurements
Chiu et al., 2013 [17]	Quantitative study	Caregivers (211)	China	Face concern	Affiliate stigma	The Loss of Face Scale (LOS) The Affiliate Stigma Scale (AAS)
Mirza et al., 2019 [12]	Quantitative study	Students (173)	White British and South Asians	Supernatural beliefs	Public stigma	The Beliefs About Mental Health Problems Questionnaire The Supernatural Attitudes Questionnaire (SAQ)
Mak et al., 2015 [18]	Quantitative study	Adults (199)	China	Face concern	Self-stigma	Loss of Face (LOF), 9-item Self- Stigma Scale
WonPat-Borja et al., 2012 [19]	Quantitative study	Chinese Americans (42) and European American (428)	the USA	Face concern and familism	Public stigma	Three items measuring eugenic attitudes and one item measuring the importance of genetics in mental health ‘three items that describe intimate social distance
Mak & Cheung, 2012 [20]	Quantitative study	Caregivers (108)	China	Face concern	Affiliate stigma	ASS, LOS
Chiu et al., 2015 [21]	Quantitative study	Caregivers (211)	China	Face concern	Affiliate stigma	ASS, LOS
Griffiths et al., 2006 [22]	Quantitative study	Adults (3998 from Australia and 2000 from Japan)	Australia and Japan	Individualism	Self-stigma and Public stigma	Perceived stigma scale; Personal stigma scale
Boge et al., 2018 [23]	Quantitative study	Adults (924)	India	Familism	Public stigma	Link’s Perceived Discrimination and Devaluation Scale (PDDS).
Picco et al., 2016 [24]	Quantitative study	Adults (280)	Singapore	Cultural values and beliefs affect stigmatization	Self-stigma	Internalized Stigma of Mental Illness; Rosenberg’s Self Esteem Scale; Dispositional Hope Scale (DHS)
Ran et al., 2018 [2]	Quantitative study	Adults (153)	China	Cultural values and beliefs affect stigmatization	Self-stigma	Internalized Stigma of Mental Illness;
Mileva, Vázquez & Milev, 2013 [25]	Quantitative study	Persons with bipolar disorder (178 from Argentina and 214 from Canada)	Argentina and Canada	Cultural values and beliefs affect stigmatization	Public stigma	Inventory of Stigmatizing Experiences (ISE)
Marquez & Ramírez, 2013 [26]	Quantitative study	Caregivers (32)	Mexican caregivers in the USA	Familismo	Affiliate stigma	Five items scale developed by Greenberg, Greenley, McKee, Brown, and Griffin-Francell (1993)
Lin et al., 2018 [27]	Quantitative study	Mental health professionals (665)	China and the USA	Confucianism and face concern	Professional stigma	The Clinician Associative Stigma Scale (CASS)
Pang et al., 2017 [28]	Quantitative study	Youths (940)	Singapore	Collectivism and face concern	Public stigma	The Attitudes Towards Serious Mental Illness; Social Tolerance Scale
Haraguchi et al., 2009 [29]	Quantitative study	Rehabilitation workers (292 from Japan and 270 from China).	China and Japan	Cultural values and beliefs affect stigmatization	Public stigma	The Social Distance Scale
Caplan et al., 2011 [30]	Quantitative study	Adults (177)	Latino immigrant in the USA	Religion and supernatural beliefs	Public stigma	Beliefs about Causes of Depression scale; Perceived stigma contained three items
Kurumatani et al., 2004 [31]	Quantitative study	Elementary school teachers (150 from Taiwan and 129 from Japan)	Taiwan and Japan	Cultural values and beliefs affect stigmatization	Public stigma	Angermeyer and Matschinge questionnaire
Papadopoulos, Foster & Caldwell, 2013 [32]	Quantitative study	4 culture groups (78 Americans, 75 White-English, 77 Greek, 75 Chinese)	White-English, American, Greek, Chinese	Individualism and Collectivism	Public stigma	Community Attitudes to Mental Illness Scale; the ‘vertical-horizontal individualism-collectivism scale
Loya, Reddy, & Hinshaw, 2010 [33]	Quantitative study	Students (74 Caucasians and 54 South Asians)	South Asian and Caucasian in the USA	Collectivism	Public stigma; Self-stigma	Social Distance Scale; Devaluation-discrimination Scale
Tsang et al., 2007 [34]	Qualitative study	Employees (40 from Chicago, 30 from Hong Kong, and 30 from Beijing)	China and the USA	Confucianism and religions	Public stigma	N/A
Tanaka et al., 2018 [35]	Qualitative study	People with mental illness (39)	Philippines	Religion and supernatural beliefs	Self-stigma	N/A
Fancher et al., 2010 [36]	Qualitative study	Adults (11)	Vietnamese American in the USA	Supernatural beliefs and familism	Self-stigma	N/A
Luo et al., 2018 [37]	Qualitative study	Medical school graduates (20)	China	Face concern	Professional stigma	N/A
Chen et al., 2013 [38]	Qualitative study	Persons with mental illness (53)	Chinese immigrant in the USA	Face concern and familism	Self-stigma	N/A
Han et al., 2017 [39]	Qualitative study	Participants aged from 18 to 49 (18)	Korean Immigrants in the USA	Collectivism and face concern	Public stigma	N/A
Ramli et al., 2017 [40]	Qualitative study	Caregivers (14)	Malaysia	Religion and familism	Affiliate stigma	N/A
Mascayano et al., 2015 [41]	Qualitative study	Persons with mental illness (20)	Chile	Familism	Self-stigma	N/A
Alvidrez et al., 2008 [42]	Qualitative study	Mental health consumers (34)	the USA	Familism	Public stigma Self-stigma	N/A
Interian et al., 2007 [43]	Qualitative study	Outpatient (30)	Latin America	Collectivism and familism	Public stigma	N/A
Caplan, 2019 [44]	Qualitative study	Persons with mental illness (177)	Latinos in the USA	Supernatural beliefs	Public stigma	N/A
Bui et al., 2018 [45]	Qualitative study	Caregivers (21)	Vietnamese in the USA	Familism and religion	Affiliate stigma	N/A
Yang et al., 2013 [46]	Qualitative study	Individuals with psychosis (20), their relatives (15), and community respondents (15).	Chile	Familism and religion	Public stigma; Self-stigma	Perceived Devaluation-Discrimination (PDD) Scale and Internalized Stigma of Mental Illness Scale (ISMI)
Keller et al., 2019 [47]	Qualitative study	Students (33)	the USA	Collectivism and individualism	Public stigma; Self-stigma	N/A
Yang, 2015 [48]	Mixed-method	Caregivers (120)	China	Face concern and familism	Affiliate stigma	ASS, LOS
Yang et al., 2014 [49]	Mixed-method	Caregivers (11)	Chinese Immigrants in the USA	Familism	Affiliate stigma	Internalized stigma scale
Lee et al., 2005 [50]	Mixed-method	Out-patients (480)	Hong Kong	Face concern	Public stigma	Psychiatric Stigma Experience Questionnaire (PSEQ)
Mascayano et al., 2016 [51]	Theory study(review)	Latin Americans	Latin America	Familism, and religion	Public stigma, self-stigma, affiliate stigma, and multiple stigmas	N/A
Yang & Kleinman, 2008 [52]	Theory study	Chinese	China	Face concern	N/A	N/A
Yang et al., 2007 [53]	Theory study	Chinese	China	Face concern	N/A	N/A
Hanzawa, 2012 [54]	Theory study	Japanese	Japan	Confucianism and familism	Affiliate stigma	N/A
Abdullah & Brown, 2011 [11]	Theory study(review)	Americans (different ethnicities)	the USA		N/A	N/A

Regarding the risk of bias, the studies were categorized into the high, moderate and low risks. Although five of the studies were theory studies, in consideration of the purposes of providing a wide review on the topic, they were included in the final review. As a result, all studies were considered to display an acceptable risk of bias.

### Stigma type

There were four types of stigma of mental illness as following: public stigma, self-stigma, affiliate stigma and professional stigma.

### Public stigma

According to the results of studies reviewed [12, 19, 23, 34, 39, 51], persons with mental illness usually were labelled as dangerous, weak, strange, incompetent and blameworthy. The perception that persons with mental illness behave violently was considered as a major reason for general public displaying stigmatizing behaviors and attitudes toward persons with mental illness. Due to exposure to prevalent public stigma, a substantial number of persons with mental illness encountered isolation, rejection and social distance from society, friends, families, and partners [39]. Stigma from close individuals, such as relatives and friends were more hurtful than that of general public [50]. Furthermore, stigmatizing beliefs and stigmatizing actions might deter help seeking and treatment, which is associated with symptom deterioration [12]. Additionally, Abdullah and Brown (2011) [11] summarized that cultural values related to social image, such as marianismo (that females should be self-sacrificing) and machismo (that males should be strong) in Latino values, also caused public stigma for persons with mental illness. Such kinds of social image also appeared in Indian culture, where the homemaker role keeps them from social support and resulted in fear of social rejection [23]. Two studies demonstrated that public stigma toward persons with mental illness might be more severe in non-Western cultures [12, 34]. A comparative study [22] summarized that although both Japanese and Australian public perceived persons with mental illness as dangerous and unpredictable, Japanese public also strongly believed that mental illness involved in personal weakness. Notably, Yang and Kleiman (2008) [52] conceived that stigma was a moral issue and “face” constructed by moral standing played an important role in Chinese societies. As a result, the moral status of persons with mental illness might be lower and they had higher difficulties accessing social capital due to loss of face.

### Self-stigma

13 studies [2, 18, 22, 24, 33, 35, 36, 38, 41, 42, 46, 47, 51] addressed self-stigma of persons with mental illness. Evidence suggested that self-stigma regarding mental health was associated with lower level of self-esteem, which in turn resulted in negative outcomes [41]. Two studies [35, 38] indicated that stigma of mental illness might reduce the social networks of persons with mental illness considerably. In particularly, evidence demonstrated that face concern was strongly associated with self-stigma [18, 36]. In a study from Chile [41], the manifestation of stigma was influenced by the role of family and social status. In short, research provides substantial evidence that cultural values may play significant roles in stigma internalization.

### Affiliate stigma

Results from 10 studies [17, 20, 21, 26, 40, 45, 48, 49, 51, 54] demonstrated that mental illness stigma did not just affect those with mental illness, but also influenced their family caregivers [17]. Apart from economic and caregiving burden, family caregivers themselves often experienced poor mental health [45]. Affiliate stigma, the stigma felt due to the mental health or disability of a loved one, contributed to this burden. Caregivers might have cognitive and behavioral reactions including social withdrawal, self-compassion, overprotection, perceptions of lower competence and worth than their peers, and feelings of shame and embarrassment [20, 21, 40, 48, 54].

### Professional stigma

Stigma also affects mental health professionals, such as psychiatrists, menta health counselors, social workers and nurses due to having a connection with persons with mental illness. An occupational hazard for mental health professionals working with those with mental illness might include experienced stigma from the public, i.e., professional stigma or associative stigma, which might also lead to professionals enacting stigma against their clients or holding negative attitudes toward their clients [27, 37]. Luo et al. (2018) [37] reflected that apart from fear of violent behavior of persons with mental illness, loss of face was another major concern for professionals that working in underdeveloped psychiatric facilities with negligent care made them feel shame and engage in lower social status. Mental health professionals might exhibit significantly different levels of social acceptance toward persons with mental illness in various sociocultural contexts.

### Rates of stigma of mental illness

Table 2 shows the rates of self stigma and public stigma toward mental illness in Pacific Rim region. Overall, the rate of stigma of mental illness in Pacific Rim region was relatively high (e.g., self stigma in persons with mental illness: from 15 to 94.7%; public stigma: from 25.4 to 85.2%), especially in China and the USA. It could be expressed by the influence of various sociocultural contexts.

**Table 2:** The Rates of Self and Public Stigma of Mental Illness in Pacific Rim Region

Study	Country/District	Sample	Type of Stigma	Assessment	Rate
Ran et al., 2018 [2]	Mainland China	453 persons with schizophrenia in rural community	Self-stigma	Internalized Stigma of Mental Illness (ISMI)	Moderate to severe: 94.7%
Picco et al., 2016 [24]	Singapore	280 adults with mental illness	Self-stigma	Internalized Stigma of Mental Illness (ISMI)	Moderate to severe: 43.6%
Alvidrez et al., 2008 [42]	USA	34 African Americans with mental illness	Self-stigma	Self-report	15–68.0%
Mileva, Vázquez & Milev, 2013 [25]	Argentine	178 adults with bipolar disorder	Self-stigma	Inventory of Stigmatizing Experiences (ISE)	36.2–61.7%
Lee et al., 2005 [50]	Hong Kong	320 out-patients with schizophrenia	Self-stigma	Psychiatric Stigma Experience Questionnaire	Work-related stigma: 40.2–46.8%; Interpersonal stigma 27.9–68%; Concealment and anticipated stigmatization: 28.8–69.7%;
Griffiths et al., 2006 [22]	Australia	3998 general adults aged over 18 years	Public Stigma	Perceived stigma scale	35.6–85.2%
	Japan	2000 general adults aged 20 to 69	Public Stigma	Perceived stigma scale	30–82% 43.5%
Boge et al., 2018 [23]	India	924 general adults in cities	Public stigma	Link’s Perceived Discrimination and Devaluation Scale (PDDS)	39.7–57.7%
Kurumatani et al., 2004 [31]	Japan	129 Japanese elementary school teachers	Public stigma	Angermeyer and Matschinge questionnaire	36.3%
	Taiwan	150 Taiwanese elementary school teachers	Public stigma	Angermeyer and Matschinge questionnaire	25.4%
Pang et al., 2017 [28]	Singapore	940 general youths from schools	Public stigma	Social Tolerance Scale	44.5%

### Cultural factors

Table 3 shows the main findings of cultural factors in the reviewed studies. The major cultural factors or values were illustrated as following.

**Table 3:** Main Findings of Cultural Factors in the Reviewed Studies

Author, year	Main findings
Tsang et al., 2007 [34]	Chinese employers were more likely to perceive that people with mental illness would exhibit a weaker work ethic and less loyalty to the company.
Tanaka et al., 2018 [35]	Fatalism could help PMHP to remain hopeful. In addition, traditional communal unity alleviated some of the social exclusion associated with stigma.
Chiu et al., 2013 [17]	Chinese caregiving was characterized by a lack of formal support, and such cultural concerned as loss of face and strong affiliated stigma.
Mirza et al.,2019 [12]	South Asians reported higher beliefs in supernatural causes of psychosis than White British.
Mak et al., 2015 [18]	Role of face concerned in affecting self-stigma and mental health among Chinese with substance use problems
Yang et al., 2007 [53]	Stigma exerted its core effects by threatening the loss or diminution of what is most at stake, or by actually diminishing or destroying that lived value.
Fancher et al., 2010 [36]	Four themes: (1) Stigma and face; (2) Social functioning and the role of the family; (3) Traditional healing and beliefs about medications; and (4) Language and culture.
WonPat-Borja et al., 2012 [19]	Chinese Americans endorsed all four eugenic statements more strongly than European Americans
Luo et al., 2018 [37]	Low levels of social acceptance of individuals with mental illness among medical students in China were largely related to fears of violence of and loss of face.
Chen et al., 2013 [38]	Participants commonly suffered from stigma after disclosure. However, half of our participants reported situations where they experienced little discriminatory treatment and some experienced support and care as a result of cultural dynamics.
Abdullah & Brown, 2011 [11]	Cultural values are important with regard to stigma, particularly for Asian Americans and African Americans.
Han et al., 2017 [39]	The study findings revealed stigmatized beliefs (e.g., being dangerous, out of control, and abnormal) and behaviors (e.g., social distance) toward people with mental illness, as well as cultural values that reinforced the stigma in the Korean-immigrant community.
Yang, 2015 [48]	Caregivers with higher face concern were more likely to internalize feelings of shame, self-blame and powerlessness and suffered poorer mental health.
Mak & Cheung, 2012 [20]	Affiliate stigma was found to serve as a partial mediator between face concern and care- giver distress and a full mediator between face concern and subjective burden.
Ramli et al., 2017 [40]	Most Malay caregivers experienced the stigma around mental health problems regardless of the type of mental illness.
Chiu et al., 2015 [21]	The mediating role of affective stigma was confirmed.
Mascayano et al., 2015 [41]	A key feature shaping stigma among females was the loss of ability to fulfill family roles (i.e. take care of children). For males, cultural value of ‘Machismo’ kept them from disclosing their psychiatric diagnosis as a means to maintain social status. This is attribute to ‘Familismo’.
Griffiths et al., 2006 [22]	Personal stigma and social distance were considerably greater among the Japanese than the Australian public, which is connected to the perception of the attitudes and discriminatory behavior of others.
Hanzawa, 2012 [54]	persons with mental illness had greater likelihood to isolate themselves, thus refusing contact with nonfamily members. Such kinds of behaviors increased caregivers’ burden. Japanese families did not allow others to care family members with mental illness.
Boge et al., 2018 [23]	Gender differences in cultural and societal roles and expectations could account for higher levels of perceived stigma among female participants. A higher level of perceived stigma among female participants was attributed to cultural norms and female roles within a family or broader social system.
Interian et al., 2007 [43]	Stigma resulted in negative social outcome and caused by cultural values.
Alvidrez et al., 2008 [42]	Concerns about stigma caused most Black Americans initially to avoid treatment; They commonly were exposed to stigmatizing reactions from others when they accepted treatment.
Picco et al., 2016 [24]	There was a negative association between quality of life and self-stigma, which may be expressed by cultural values and beliefs.
Ran et al., 2018 [2]	Self-stigma among persons with mental illness was pervasive and severe in rural community in China. Ongoing evaluation and measurement of stigma in the Chinese context would play a crucial role in understanding culture-specific aspects of experiencing self-stigma.
Yang & Kleinman, 2008 [52]	Stigma was embedded in the moral experience of participants across culture.
Mileva, Vázquez & Milev, 2013 [25]	People with bipolar disorder experienced stigma and psychosocial effects. Canadian and Argentinean societies showed different family dynamics due to diverse cultures.
Marquez & Ramírez, 2013 [26]	Familism, folk beliefs, and shame might result in Latinos’ lower service usage. Caregivers reported that cultural beliefs acted as barriers to mental health service use among Latinos
Lin et al., 2018 [27]	Professional stigma was considerably lower in China than in the US, possibly indicating the cultural dominance of respect for professionals over stigma towards persons with mental illness.
Caplan, 2019 [44]	The cultural values contributed to shaping stigma but also could be an important source to cope mental illness.
Bui et al., 2018 [45]	Religion offered an important coping strategy to persons with mental illness. Mental health education and use of less stigmatizing language might facilitate early intervention by reducing stigma.
Pang et al., 2017 [28]	The contexts of stigma and social tolerance were different between Asian cultures and Western cultures. Chinese youths displayed higher level of ‘physical threat’ and lower level of ‘social tolerance’ than their counterparts of other ethnicities.
Yang et al., 2014 [49]	This study pointed out an initial but crucial approach to reduce stigma of mental illness among Asian Americans who influenced by stigma powerfully role.
Lee et al., 2005 [50]	Stigma was common, hard to prevent and devastating to people with schizophrenia. Family support was required to be realized with the emphasis on relationship bonds in Chinese societies.
Haraguchi et al., 2009 [29]	Social distance towards schizophrenia was widely common in both Beijing and Fukuoka, but the features of social distance was not similar between them.
Caplan et al., 2011 [30]	Latino immigrants strongly endorsed that depression was caused by both malevolent spiritual forces and psychosocial issues, reflecting that they engage in a dual system of Western-medicine and spiritual beliefs.
Yang et al., 2013 [46]	Stigma of mental illness endangered the males’ ability to protect their family honors, and the females’ ability to become holy and pure. What’s worse, it further threatened the family ability. Development of culture-specific stigma measures played an important role in implementation of community mental health care in Latin American contexts.
Keller et al., 2019 [47]	Individualist orientation was more common for Caucasians, collectivist orientation was more common for Native Americans, indicating that it was necessary to address culture difference during the process of formulating programme to reduce stigma of mental illness.
Kurumatani et al., 2004 [31]	Japanese and Taiwanese displayed similar knowledge, beliefs and attitudes with regard to schizophrenia with the general public in Western countries.
Papadopoulos, Foster & Caldwell, 2013 [32]	Individualism contributed to more positive attitudes towards mental illness, while collectivism contributed to more stigmatizing attitudes towards mental illness.
Loya, Reddy, & Hinshaw, 2010 [33]	The South Asian students displayed greater personal stigma towards mental illness than Caucasian students, which might be influenced by South Asians cultural values which emphasize a collectivist orientation and a hierarchical and family structure.
Mascayano et al., 2016 [51]	Stigma was common across cultures and influenced by cultures profoundly. There was significantly local difference regarding stigma in Latin American contexts.

### Confucianism and collectivism

Confucian and Collectivist values were examined by several studies. Abdullah and Brown (2011) [11] demonstrated that many East Asian cultures were deeply rooted in collectivist and Confucian values, which emphasize harmonious social relationship and solidarity, especially in Chinese, Japanese and Korean communities. Individuals were required to adhere to the norm and those with inappropriate behavior were often disparaged publicly in these communities [28, 37]. In general, mental illness is considered a strain on collective systems within Asia, including families, social groups and society as a whole because of the common stereotypes that persons with mental illness are strange, dangerous, unpredictable and out of control. Many Asians might display stigmatizing behavior toward persons with mental illness, such as declining to hire them [34, 39]. Pang et al. (2017) [28] found that Chinese youths with mental illness suffered from more physical threat and higher level of social distance due to influence of collectivism. Even in some Asian countries, individuals with mental illness might be viewed as representing the family’s mental illness, bad blood, or past misdeeds [11, 17, 29, 52, 53].

Apart from Asian societies, Latino Americans, Native Americans and African Americans also engaged in collectivism and cooperation [42, 47], which is contradictory to Caucasian Americans that mainly value individualism. In contrast, Caucasian people might be less likely to pay attention to whether individuals have harmonious relationships. For example, compared with their counterpart in China, Caucasian American employers concerned more whether their employees had the required skills to finish the job rather than placing value on social relationships [34]. This might be that Caucasian Americans tend to consider mental illness as an individual experience in nature [47]. Consequently, Caucasian American employees might suffer from less stigma [34].

### Face concern and familism

Face concern, which refers to individuals’ desire to maintain their social image, social values and social capitals influenced by their performance and specific social roles, has been well documented in collective societies [21]. Yang and Kleinman (2008) [52] illustrated the stigma regarding moral experience in China. Generally, face concern in Chinese culture consists of two dimensions: *lian* (moral worth) and *mianzi* (social values) in Chinese culture [17, 18]. Face concern is associated with the feeling of shame and internalization of stigma. Persons with mental illness and their families in some Asian communities, such as Chinese, Korean, Japanese and Vietnamese, tended to avoid disclosure of mental illness in order to save face, which might lead to delayed treatment seeking. This might be because they held the belief that maintaining a respectable standing in their social network was essential [39, 53], and mental illness was associated with poor moral character, spiritual issues, or family issues [36, 45, 52]. Furthermore, loss of face might result in several negative consequences, such as being classified as an outcast and having difficulties maintaining a good reputation [18]. In the cultural context of Latin America, persons with mental illness might feel that they were not worthy of dignity and respect, which is linked to the process of self-stigma [11]. With respect to public stigma, it was found that the general public concerns whether interactions with persons with mental illness might lead to a loss of face [37].

Given that persons with mental illness were commonly regarded as a shame or guilt to the whole family among Chinese, Korean, Japanese and Latino, family caregivers with strong face concern might also experience heightened psychological stress [21]. Some chose to seek support from family instead of seeking formal medical treatment [36, 46]. Japanese family caregivers even resisted persons with mental illness interacting with people outside the family. Interian et al. (2007) [43] found that Latinos with family-orientation had a greater likelihood of refusing diagnosis and treatment. In the context of Latin American, the traditional gender value “machismo” contextualized within the framework of the culture known as familism also played a powerful role in shaping stigma of mental illness, given that machismo states male’ ability to protect family honor, and the female’s ability to be a proper and pure mother, while suffering from mental illness is considered as failing to fulfill their family obligation [51].

The importance of the link between genetics and mental illness might lead to parents and family members disapproving of marriage with persons suffering mental illness [35, 37, 52]. Compared with European Americans, Chinese Americans paid more attention on heredity of mental illness [19]. Actually, evidence in relation to familism also showed that African Americans considered mental illness as a family’s failure to tackle the issue [42].

Face concern and familism play significant roles in stigma of mental illness in collective cultures of the Pacific Rim region. Yang et al. (2007) [53] also explored the moral values in American cultures, but did not provide adequate support that stigma possess a moral dimension within European and North American traditions. Further studies should be conducted in this area.

### Religion and supernatural beliefs

Although Western treatment methods for mental illness, such as medication, were considerably widespread in Asian and Latin American societies, supernatural factors, including traditional and folk religion were still endorsed in Vietnam, India, China and Latin districts, especially in rural areas [12]. Many people strongly believed that mental illness involved evil spirits, fate, punishment prompted by God, ancestors or dead souls [34, 35, 45]. Abdullah and Brown (2011) [11] also reported that many Asian Americans might be more likely to believe that mental illness is a punishment caused by God due to previously bad behavior. For example, Vietnamese Buddhists believed the cause of mental illness lied in “karma”, such that persons with mental illness must had done bad things in their past life [45]. Many Chinese people considered that possible reasons of mental illness might be related to problems of ancestor’s tomb [2].

Latino Americans who engaged in Catholicism conceptualized mental illness as resulting from lack of faith, without praying and devil occupying mind, or demons [44]. They pervasively attributed ill to God’s will, suggesting the presence of fatalistic views [30]. Many persons with mental illness consequently visited spiritual healers or temples for their initial consultation regarding a possible mental health concern [12, 28, 44].

### Individualism

Abdullah and Brown (2011) [11] concluded that most research regarding stigma of mental illness in the USA used the samples of Caucasians who influenced by European background and values. The most significant value of that was individualism and competition which rooted in independence, autonomy and individual success. In line with others ethnic groups, stigma of mental illness also deterred Caucasian with mental illness from treatment [11, 22], which might be explained by their concern for individual success and constant comparison to the others. Papadopoulos et al. (2013) [32] compared the difference regarding stigma among four cultural groups, including a White-English group, a Greek Cypriot group, a Chinese group and an American group, indicating that individualism which affecting American group profoundly was associated with less stigmatizing attitudes towards mental illness. One possible mechanism was that individualist cultures was highly relevant to tolerate diversity and deviation from social norms. Apart from that, mental illness was described as an individual issue, especially amongst Caucasians [47].

### Stigma measurements

There were numerous measurements to assess different type of cultural factors related to stigma of mental illness, such as Loss of Face Scale (LOF) [55] and Supernatural Attitudes Questionnaire (SAQ) [56] (Table 1). In a Chilean study [46], “culture-specific” module was considered to add in two significant stigma measures to construct a psychometrically validated tool. Given the fact that the meanings, practices, and outcomes of stigma of mental illness are varied across culture, it is necessary to obtain perspectives from different constituencies in order to fully understand how stigma affects within particular sociocultural context. Multiple methodologies, including ethnographic methods and other several supplemental methodologies, e.g., art-based research methods such as a community-based theatre [47] and poetry [57] might work effectively to address how to measure stigma of mental illness in specific contexts [53].

### Anti-stigma interventions

Yang et al. (2014) [49] conducted and evaluated a pilot anti-stigma intervention with 3 sessions including psychoeducation, countering experienced stigma and countering internalized stigma among Chinese caregivers with affiliate stigma. They pointed out that it was important to address cultural concern with flexibility during the process of implementing intervention whatever apply any advanced or effective model. Additionally, several studies provided suggestions to develop effective anti-stigma strategies. Ramli et al. (2016) [40] stated that the intervention should meet several requirements, including being community-based, carefully targeted, and culturally specific. Yang (2015) [48] and Chiu et al. (2015) [21] demonstrated the intervention could be conducted at three levels: personal/individual level, family/interpersonal level, and community/societal level. At the personal level, it was essential to alleviate self-stigma and reduce the burden for persons with mental illness and caregivers, as well as emphasize social functioning. Intervention such as “re-moralization” counseling could help restore the face and re-engage society [20, 21]. At the family/interpersonal level, positive communication, network-based interventions, and caregiving task sharing were effective [36, 38, 48]. Furthermore, a systematic and scientific health care system and welfare service provision toward persons with mental illness were crucial at the community/societal level, such as the Mental Health Act introduced in 2018 contributed to reducing stigma and protecting the rights of persons with mental illness in Philippines [35]. One of significant strategies was increasing public understanding of mental illness [35], which is consistent with other studies on developing psychoeducation programs [12, 38, 42]. Mass media might serve as an effective tool to shape public perception and attitudes toward mental illness [37]. However, there were no studies to explore the effectiveness, especially long-term effectiveness, of the enhancing contact with persons with mental illness on reducing stigma of mental illness. Further studies should be conducted in this important area.

## Discussion

To our knowledge, this is the first study to review the cultural impact on stigma of mental illness in the Pacific Rim region. This study should be crucial for promoting culture-specific mental health services and interventions for reducing stigma toward persons with mental illness and their relatives, considering that stigma is a significant barrier to recovery. This systematic review identified key studies conducted within the Pacific Rim region on stigma of mental illness and cross-cultural characteristics with combination of both quantitative and qualitative studies.

The present review explored the multiple stigma of mental illness. In accordance with a previous study [3], the majority of the public perceive persons with mental illness as unpredictable and dangerous, which causes persons with mental illness to suffer from social distancing, exclusion and isolation. One of the significant negative results is to impede professional help seeking, which is not only driven by public stigma, but also self-stigma, and affiliate stigma. Disclosure and confidentiality concerns are common among persons with mental illness and their relatives due to the impact of cultural factors or values [28]. Empirical findings [27, 37] also showed that mental health professionals might display stigma towards their patients, which can be explained by the perception of difficulties interacting with them, and reflects the relatively low levels of social acceptance toward persons with mental illness. Even physicians who work in the mental health field might also experience misunderstanding and suspicion by their families and general public [37]. What’s worse, evidence indicated that professional stigma was negative associated with job satisfaction and quality of treatment [58].

Compared with the rates of stigma of mental illness in other countries, such as 45% Turkey, 27.4% Israel and 58% Poland [8], the rates of stigma of mental illness (e.g., public stigma) are relatively higher in the Pacific Rim region as noted earlier. The interpretation might lie in the traditions and values of individualism. However, it is noteworthy that prevalence of stigma of mental illness among China and USA exhibit great diversity among different studies because of disparities in socioeconomic development, ethnicities across different states and provinces, and various assessments. Currently, the prevalence of stigma has been widely explored in European studies, further studies on the rate of stigma of mental illness should be conducted in the Pacific Rim region, especially many small countries. The standard measurements of stigma of mental (e.g., self stigma and public stigma) illness should be used in the future studies.

Countries and districts in the Pacific Rim region exhibit significant cultural differences towards stigma of mental illness. In Asia and Latin America, collectivism and familism have profound effects that shape stigma towards mental illness. In addition to face concern and familism, East and South Asians, such as rural China, India, Malaysia and Singapore are also more likely to be influenced by supernatural factors and folk religion. However, the stigma experienced by Americans is not similar due to widely diverse ethnicities [11]. The Pacific Rim region can be observed a mixture of extensive culture [59].

In terms of culture and stigma of mental illness, East Asians, such as Chinese, Japanese and South Koreans, are seriously affected by Confucianism and collectivism which emphasizes social harmony and individual’s duty to follow social norms and maintain order [10, 54, 60]. Persons with mental illness are frequently seen as abnormal and erratic by Confucianism and collectivism, which leads to stigma beliefs and discriminative actions toward persons with mental illness by general people [39]. The results of this review also indicate that persons with mental illness and their relatives in collective societies have to struggle with more severe and widespread stigma than their counterparts in Western societies [34].

The most commonly illustrated theme emerging from this review was face concern and familism. In the culture context of Asian, Latinos and African Americans, people often take special care of face and family reputation. Face plays an important role in East Asian and Latino cultures regarding stigma of mental illness, and not only affects persons with mental illness, but also families, since that mental illness is recognized as shame and disease of the whole family. In other words, they may attribute mental illness more or less to family issue [21, 35]. Those with strong face concern are more vulnerable to self-stigmatization, therefore they feel it is essential to avoid disclosure of mental illness, which in turn inhibits persons with mental illness from seeking professional service [18]. Moreover, evidence also suggested that face did not affect stigma of mental illness alone [17, 27].

The findings of this review also suggest that religious and supernatural beliefs have significant impacts on people’s beliefs and behaviors toward mental illness in Latin America and Asia, such as the Bayanihan spirit for Tagalog. One interpretation is that they are more religious and tend to attribute mental illness to supernatural reasons, especially in the rural areas [2, 12, 35]. However, further studies should be conducted to explore the impact of supernatural beliefs on stigma of mental illness.

The studies aforementioned explain how cultural factors affect to increasing stigma of mental illness. However, the effect of cultural factors or values are two-sides that it also might contribute to reducing stigma. Caplan (2019) [44] explained that religion could be powerful sources for Latinos with mental illness, given that churches might offer spiritual and educational resource to persons with mental illness. Influenced by Christian religions, compassionate and humane care were endorsed by mental health professionals, which contributes to reducing stigma towards persons with mental illness [51]. Additionally, in a Vietnamese case, Buddhism helps persons with mental illness and their caregivers to understand mental illness. Some caregivers might be proud of providing care because they consider the experiences fulfill their religious obligation “Karma” [45].

Thus, it is possible that some kinds of religious or positive spiritual beliefs are important source for persons with mental illness and their relatives to against stigma. More importantly, though Confucian values and familism might cause that persons with mental illness experience isolation from their families and communities, the emphasis on sharing of responsibilities and family obligations to provide care persons with mental illness and the relatives, which may also contribute to buffering the negative effect of stigma because family involvement and support could be a positive attribute to recovery [39, 40, 45]. It is evident that cultural factors or values operate stigma in different ways. Further studies need to elaborate more in what condition these cultural factors or values contribute to reducing the stigma and helping persons with mental illness and their relatives.

With regard to anti-stigma intervention, there is limited study to evaluate the effectiveness, especially long-term effectiveness, of such intervention. Several studies in this review provided anti-stigma strategies and implication for practice based on their results. The findings support that implementation of a culture-specific, carefully targeted and comprehensive intervention at personal/individual level, family/interpersonal level or community/societal level, significantly contributes to reduction in stigma for diverse groups. For example, taking account of face concern to address self-stigma driven by fear of loss of face, counselors and social workers are suggested to provide “re-moralization” intervention to restore the face [20, 52]. Such kinds of interventions to Chinese immigrant incorporating cultural notion “face” closely provided a pattern and might work effectively to resist stigma among other Asian ethnic groups [49], such as South Koreans who also pay particularly close attention to face-saving, i.e., “chemyun” [39]. Further well-designed intervention studies for persons with mental illness and their relatives should be conducted on reducing stigma of mental illness in the Pacific Rim region.

Apart from psychotherapeutic intervention and professional care towards persons with mental illness, sufficient family support for persons with mental illness and caregivers to reduce their stress and promote their mental health is also essential. At the societal level, community-based care systems and systematic social policy are essential to empower them and to prevent them from internalization of stigma and empower them [2, 35]. Specifically, mental health education has been widely emphasized. Luo et al. (2018) [37] indicated that such kinds of education through media had adequate influence to shape public attitude and perception toward mental illness. According to Griffiths et al. (2006) [22], the reason that Australians display lower level of self-stigma than Japanese might lies in the fact that Australia emphasized the community-based rehabilitation services and widespread public health education. It is worth mentioning that media should be in charge of decreasing stigma of mental illness in some societies because of its power and ability to frame persons with mental illness in a positive light. Except persons with mental illness and their relatives, the stigma towards mental health professionals also should be tackled, given that such kind of stigma is associated with their job stress and quality of treatment to persons with mental illness [58].

In terms of specific measures for stigma of mental illness within a cultural perspective, Yang et al. (2007) [53] highlighted the importance of multiple methodologies given the fact that the stigma experience varies in different societies and situation, which is in accordance with a previous study [10]. In the study carried out by Mirza et al. (2019) [12], the Beliefs About Mental Health Problems Questionnaire (BAMHPQ) utilized to measure biological, psychosocial, and spiritual causes was reported lower reliability and failure of capturing cultural differences. One possible explanation could be differences between particular sociocultural contexts. Zane and Yeh (2002) [55] found that a cultural bias is common in psychotherapy agencies for assessment and treatment due to lack of culture-sensitive measurements based on clients’ experiences and culture context. Further studies should be conducted to develop more culture-specific measurements on stigma of mental illness and cultural factors.

All included provided consistent evidence that cultural values affect the process of stigmatization and anti-sigma strategy. Nevertheless, caution is required to interpret the findings, given that some studies involved qualitative research designs and methodological limitations existed in most of the studies. Additionally, it is noteworthy that the tools assessing stigma varied among different studies.

This review has certain limitations. First, most studies in which cross-country difference were carried out on the culture and value context of Asia and the USA. However, there was scant research on stigma of mental illness conducted among other countries in the Pacific Rim region, especially those in Latin America and Australia. Second, few studies focused on stigma of mental illness in some important sub populations such as elders, youths, Muslim and people with bipolar disorder and Alzheimer’s disease. Third, the sample size was relatively small in a number of the selected quantitative studies. Fourth, different measures of stigma of mental illness were used in various studies. Fifth, unpublished studies which meet all the inclusion criteria were not included, this may restrict the accuracy of the present review. Sixth, the data extraction process might have biases. Seventh, in spite of representing a diverse set of countries, the review did not include published study in languages other than English. This may cause the restriction of the cross-cultural generalizability of the findings.

Further systematic review is required to provide specific emphasis on stigma of mental illness in under-researched countries such as Thailand, New Zealand, Australia and other small countries (e.g., Pacific islands) in this region. Moreover, this is also a need for a more comprehensive analysis of the role of culture regarding stigma perceived by various groups, such as youths, LGBT people, refugees/immigrants, suicide attempt survivors, and people with other religious backgrounds and specific mental illness. Lastly, future research should explore the effectiveness of anti-stigma intervention and related strategies.

## Conclusions

This study firstly reviews the impact of cultural factors on stigma of mental illness in the Pacific Rim region. The results of this study showed that culture factors (e.g., Collectivism, Confucianism, face concern and familism, religion and supernatural beliefs) contributed to people’s stigmatizing behaviors and attitudes toward persons with mental illness, their relatives and mental health professionals. This study should be crucial for promoting culture-specific mental health services and interventions for reducing stigma toward persons with mental illness and their relatives, considering that stigma is a significant barrier to recovery. Further research on stigma of mental illness and culture-specific interventions to reduce the stigma should be conducted in the Pacific Rim region.

## Data Availability

All citations identified are in the public domain. The datasets used during the current study are available from the corresponding author on reasonable request.

## Abbreviations

AAS: Affiliate Stigma Scale
BAMHPQ: Beliefs About Mental Health Problems Questionnaire
CASS: Clinician Associative Stigma Scale
DHS: Dispositional Hope Scale
ISE: Inventory of Stigmatizing Experiences
ISMI: Internalized Stigma of Mental Illness Scale
LOF: Loss of Face Scale
PDDS: Perceived Discrimination and Devaluation Scale
PSEQ: Psychiatric Stigma Experience Questionnaire
SAQ: Supernatural Attitudes Questionnaire
